# Structural flexibility of human α‐dystroglycan

**DOI:** 10.1002/2211-5463.12259

**Published:** 2017-07-17

**Authors:** Sonia Covaceuszach, Manuela Bozzi, Maria Giulia Bigotti, Francesca Sciandra, Petr Valeryevich Konarev, Andrea Brancaccio, Alberto Cassetta

**Affiliations:** ^1^ Istituto di Cristallografia CNR, Trieste Outstation Italy; ^2^ Istituto di Biochimica e Biochimica Clinica Università Cattolica del Sacro Cuore Roma Italy; ^3^ Istituto di Chimica del Riconoscimento Molecolare CNR c/o Università Cattolica del Sacro Cuore Roma Italy; ^4^ School of Biochemistry University of Bristol UK; ^5^ Shubnikov Institute of Crystallography of Federal Scientific Research Centre “Crystallography and Photonics” of Russian Academy of Sciences Moscow Russia; ^6^ National Research Centre “Kurchatov Institute” Moscow Russia

**Keywords:** conformational stability, muscular dystrophy, small‐angle X‐ray scattering, structural flexibility, X‐ray crystal structure, α‐Dystroglycan

## Abstract

Dystroglycan (DG), composed of α and β subunits, belongs to the dystrophin‐associated glycoprotein complex. α‐DG is an extracellular matrix protein that undergoes a complex post‐translational glycosylation process. The bifunctional glycosyltransferase like‐acetylglucosaminyltransferase (LARGE) plays a crucial role in the maturation of α‐DG, enabling its binding to laminin. We have already structurally analyzed the N‐terminal region of murine α‐DG (α‐DG‐Nt) and of a pathological single point mutant that may affect recognition of LARGE, although the structural features of the potential interaction between LARGE and DG remain elusive. We now report on the crystal structure of the wild‐type human α‐DG‐Nt that has allowed us to assess the reliability of our murine crystallographic structure as a α‐DG‐Nt general model. Moreover, we address for the first time both structures in solution. Interestingly, small‐angle X‐ray scattering (SAXS) reveals the existence of two main protein conformations ensembles. The predominant species is reminiscent of the crystal structure, while the less populated one assumes a more extended fold. A comparative analysis of the human and murine α‐DG‐Nt solution structures reveals that the two proteins share a common interdomain flexibility and population distribution of the two conformers. This is confirmed by the very similar stability displayed by the two orthologs as assessed by biochemical and biophysical experiments. These results highlight the need to take into account the molecular plasticity of α‐DG‐Nt in solution, as it can play an important role in the functional interactions with other binding partners.

**Abbreviations**
DG: dystroglycan
*D*_max_: maximum size of the particle
DSF: differential scanning fluorimetry
ECM: extracellular matrix
EOM: ensemble optimization method
hα‐DG‐Nt: human α‐DG‐Nt
Ig‐like: immunoglobulin‐like
LARGE: like‐acetylglucosaminyltransferase
MMexp: experimental molecular mass of the solute
mα‐DG‐Nt: murine α‐DG‐Nt
NSD: normalized spatial discrepancy
*R*_g_: radius of gyration
rmsd: root mean square deviation
S6 domain: small subunit ribosomal protein S6 of *Thermus thermophilus*
SAXS: small‐angle X‐ray scattering
*T*_m_: melting temperatures
*V*_p_: excluded volume of the hydrated particle
α‐DG‐Nt: N‐terminal region of α‐DG
α‐DG: α‐dystroglycan
β‐DG: β‐dystroglycan

Dystroglycan (DG) is a heterodimeric transmembrane glycoprotein that is a part of the multimeric dystrophin–glycoprotein complex and plays a crucial role in the association of cells with the basement membranes 1. DG links the basal lamina with the cytoskeleton by bridging the intracellular dystrophin to a plethora of extracellular matrix (ECM) proteins, that is, laminin, agrin, and perlecan, thus offering stability to tissues. DG is highly expressed in skeletal muscle where it was first discovered 2 and where it confers structural stability to the sarcolemma during contraction, but it is also strongly expressed in heart, in brain, and in peripheral nerves, where it is involved in various physiological processes 3. Moreover, DG has been also associated with Old Word arenaviral infections, acting as a receptor for virus anchoring 4.

Dystroglycan is encoded by a single gene (*DAG1*) 2 and the corresponding precursor is proteolytically cleaved within the endoplasmic reticulum, resulting in the formation of the extracellular α‐dystroglycan (α‐DG) and the transmembrane β‐dystroglycan (β‐DG). In their mature forms, α‐ and β‐DG are linked together through noncovalent interactions involving the N‐terminal and the C‐terminal regions of β‐DG and α‐DG, respectively. α‐DG undergoes a complex, and is still not fully understood, glycosylation post‐translational process, which involves several enzymes at various stages of α‐DG maturation 5 in both endoplasmic reticulum and Golgi apparatus. A correct α‐DG glycosylation has been shown to be critical for its physiological functions. In a family of neuromuscular diseases called secondary dystroglycanopathies, the hypoglycosylated forms of α‐DG, resulting from defective enzymes responsible for α‐DG glycosylation, display limited binding capabilities toward laminin with severe implications for health 6. A low degree of α‐DG glycosylation has also been found in rare diseases caused by single point mutations hitting the DG gene 7, 8, 9. In recent years, it has been discovered that the Ca^2+^‐dependent interaction of α‐DG with its main binding partner in ECM, the LG domain, is specifically mediated by a novel polysaccharide 10. The α‐DG glycosylation biosynthetic pathways involve a kinase and several glycosyltransferases 5, among which the bifunctional glycosyltransferase like‐acetylglucosaminyltransferase (LARGE) that adds the repeating disaccharide unit (‐α3‐GlcA‐β3‐Xyl‐) to a glycan anchored at the site defined by Thr317 and Thr319 11. Indeed, it has been reported that the elongation of the glycan operated by LARGE requires the presence of the N‐terminal region of α‐DG (α‐DG‐Nt), which is thought to act as a recognition site for LARGE before being processed by a furin‐like proprotein convertase 12, 13. It has been proposed that α‐DG‐Nt would be able to bind other partners in the ECM 14, but the biological implications of these potential interactions remain elusive 15.

We aimed at characterizing the biophysical and biochemical bases behind the biological function of α‐DG, and our efforts have been focused on the comprehension of the molecular determinants that modulate its binding abilities 16. An electron microscopy study showed that α‐DG assumes a dumbbell‐like shape, with two globular N‐terminal and C‐terminal regions at the extremes of a mucin‐like region 17. Furthermore, the crystal structure of the murine N‐terminal region disclosed a modular architecture composed by an immunoglobulin‐like (Ig‐like) domain and a second domain similar to the small subunit ribosomal protein S6 of *Thermus thermophilus* (S6 domain) 18. Although the experimental structure of the C‐terminal region of α‐DG is still unknown, homology modeling suggests that its fold is likely to be a second Ig‐like structure 19. According to the crystallographic structure, murine α‐DG‐Nt (mα‐DG‐Nt) assumes an overall rather compact fold, with the Ig‐like and S6 domains interacting with each other and linked together by a flexible loop 18. Such an organization has been observed in the crystal structure of the murine α‐DG missense pathological mutant T190M, which displays the same fold, mutual orientation, and interaction between the Ig‐like and S6 domains observed for WT α‐DG‐Nt 20. Despite such high degree of structural similarity, it has been proposed that T190M might have a reduced ability to assist LARGE in its glycan elongation action, and the resulting α‐DG hypoglycosylation leads to a form of limb‐girdle muscular dystrophy 7. The comparison of the WT and T190M crystallographic models ruled out any effect of the T190M mutation on the Ig‐like and S6 domains folding, as earlier proposed by a computational study 21. Therefore, we considered whether the murine crystallographic structure could be reliably used as a general structural model for α‐DG‐Nt. In addition, we have explored α‐DG‐Nt propensity for plasticity in solution by small‐angle X‐ray scattering (SAXS) analysis of both murine and human proteins. The high‐resolution crystal structure of human α‐DG‐Nt (hα‐DG‐Nt) has also been determined, in order to assess the structural similarities between the two orthologs, which are 93% identical in their amino acid sequence. The combination of the high‐ and low‐resolution structural data (respectively, in the crystals and in solution) with biochemical and biophysical experiments proves that the murine and human proteins share a highly conserved overall architecture as well as a striking parallel structural flexibility in solution.

## Results

### Conformational stability of the N‐terminal domains of murine and human α‐dystroglycan

Conformational stabilities of murine and human α‐DG‐Nt were compared by biochemical and biophysical experiments, that is, differential scanning fluorimetry (DSF) and limited proteolysis assays.

In the DSF assay, thermally induced protein denaturation is monitored by the increase in SYPRO Orange fluorescence upon exposure of hydrophobic patches during protein unfolding. The comparison of the resulting thermal unfolding profiles and melting temperatures (*T*_m_) can be used to infer differences in the conformation and therefore in the thermal stability of the proteins 22.

**Figure 1:**
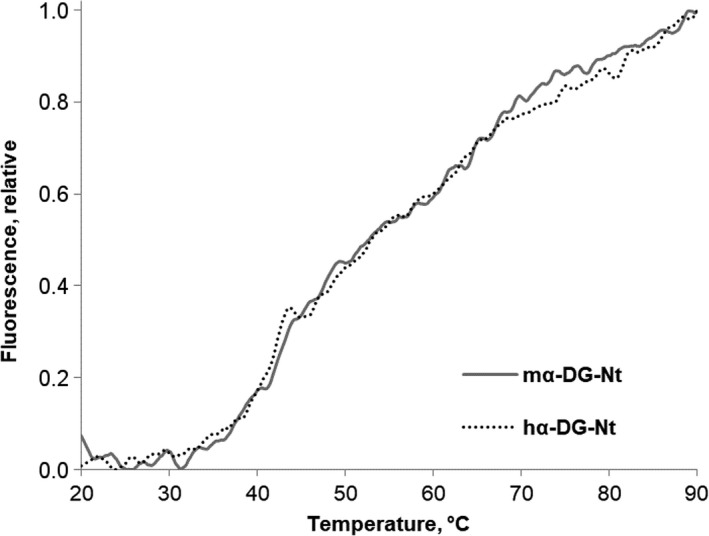
Thermal denaturation assay by DSF. Comparison of the thermal unfolding curves for murine and human α‐DG‐Nt. The increase in fluorescence emission at 516 nm indicates the association of SYPRO Orange with exposed hydrophobic residues as the protein unfolds. Experiments were performed in triplicate: A single representative curve is shown for clarity.

Figure 1 shows the changes in fluorescence emission of the murine and human proteins upon thermal unfolding in the presence of the dye.

Both the murine and human variants showed very low background fluorescence in the pretransition region that is quite flat. Their denaturation curves are almost superimposable and suggest a two‐transition unfolding process typical of protein containing two independently folded domains. The *T*_m_ values for the first transition obtained from the Boltzmann sigmoid fitting of the data (43.76 ± 0.08 °C and 43.94 ± 0.11 °C for murine and human variants, respectively) did not show any significant difference, supporting the hypothesis that the two proteins share a very similar conformational stability in solution. A similar thermal stability of the two proteins is also clear from the inspection of the second transition, although in this case the *T*_m_ could not be calculated because it was not possible to reach the aggregation region even increasing the final temperature to the upper range value of the instrument.

Limited proteolysis was also used to probe conformational stability, assuming that proteolytic recognition sites become accessible upon unfolding. Indeed, this technique is widely used to examine flexible and exposed regions, considering that proteolysis occurs exclusively at ‘hinges and fringes’ 23 and conformational parameters such as accessibility and segmental mobility correlate quite well with exposed proteolytic sites 24. Limited proteolysis experiments (data not shown) with a panel of seven different proteases did not reveal any significant difference in conformation stability or flexibility between mα‐DG‐Nt and hα‐DG‐Nt.

### Crystallographic structure of human α‐dystroglycan N‐terminal domain

The crystallographic structure of hα‐DG‐Nt has been determined at a resolution of 1.8 Å. Upon completion of the crystallographic refinement, the final *R*‐_factor_ was 0.163 (*R*‐_free_ = 0.195), with residues 52–60, 163–179, and 305–315 missing in the final model as no reliable electron density could be detected for these regions. A lower‐quality electron density was also observed for the flexible regions encompassing residues 89–91 and 181–185. The region comprising residues 159–162 shows signs of multiple conformations, but any attempt to model it during the refinement did not improve the 2*F*_o_–*F*_c_ and *F*_o_–*F*_c_ density maps, nor the refinement quality indicators.

The Ig‐like domain (residues 62–160) and the S6 domain (residues 182–305) assume the same relative orientation as observed in mα‐DG‐Nt 18 (Fig. S1A,B) with a root mean square deviation (rmsd) between the murine and hα‐DG‐Nt crystallographic models equal to 0.468 Å (calculated on 225 common *C*_α_ *s*).

Differences between the primary structures of human and murine α‐DG (see Table 1 for alignment) were easily identified in hα‐DG‐Nt, according to the 2F_o_‐F_c_ and F_o_‐F_c_ density maps (see Fig. S2 for selected examples).

**Table 1:**
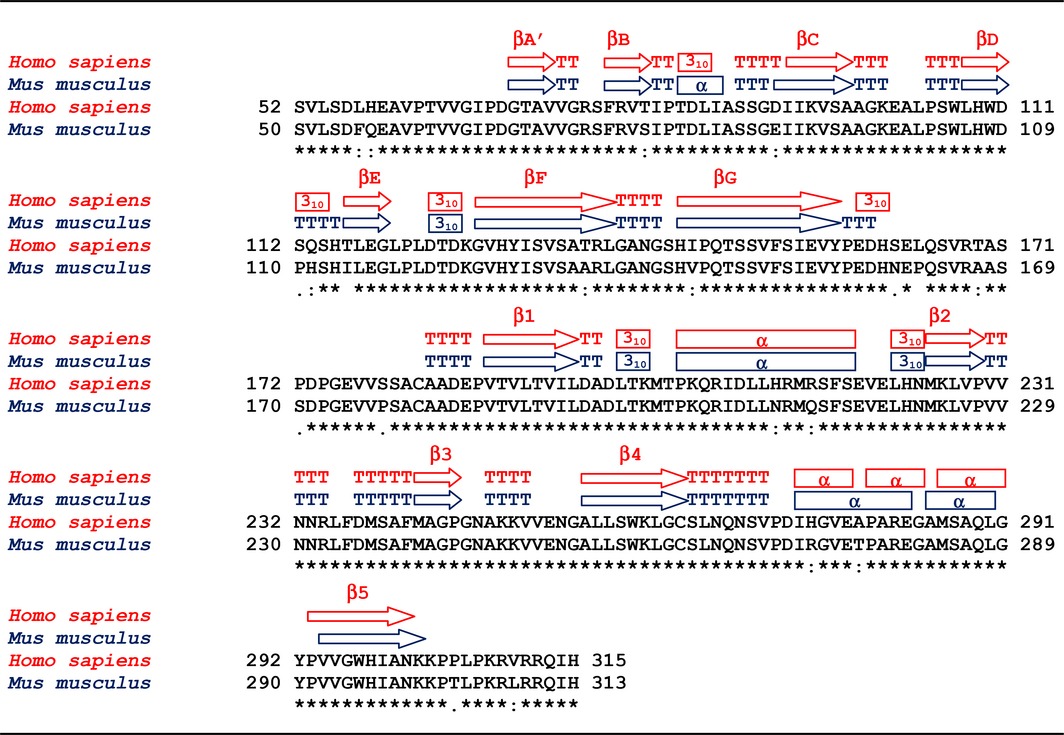
Sequence alignment

**Figure 2:**
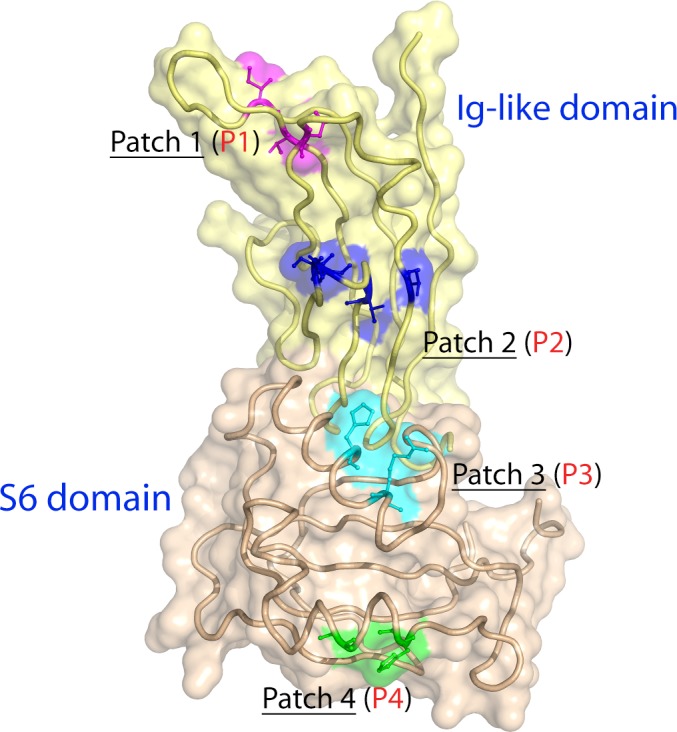
P1–P4 mapping on hα‐DG‐Nt structure and accessible surface. The hα‐DG‐Nt model is depicted as ribbon representation, with the Ig‐like and S6 domains colored in pale yellow and in pale pink, respectively. Residues belonging to the patches P1‐P4 are depicted as a stick‐and‐ball model, and their solvent‐accessible surfaces are mapped on the hα‐DG‐Nt surface. Residues and surfaces belonging to different patches are colored differently.

The differences in the primary structures are all mapped on the protein surface of hα‐DG‐Nt (Fig. 2 and Fig. S3), with residues fully or partially exposed to the bulk solvent.

The residues that are different in hα‐DG‐Nt and mα‐DG‐Nt are clustered in four small patches (Table 2) that are longitudinally distributed along one edge of the proteins (Fig. 2). Such an uneven distribution in α‐DG‐Nt seems to be an intrinsic property of this protein region (Fig. S4). Patches P1 and P2 are located on the Ig‐like domain, whereas patches P3 and P4 are on the S6 domain (Fig. S3). Patches P2 and P3 face each other in a large cleft lined by the β‐strands B, D, and E of the Ig‐like domain and by the α‐helices H2 and H3 of the S6 domains (see 18 and Fig. S3B). P2 and P3 patches do not show any explicit mutual interaction.

**Table 2:** Amino acid differences between mα‐DG‐Nt and hα‐DG‐Nt

Patch 1 (P1)			Patch 2 (P2)			Patch 3 (P3)			Patch 4 (P4)		
Res	mα‐DG‐Nt	hα‐DG‐Nt	Res	mα‐DG‐Nt	hα‐DG‐Nt	Res	mα‐DG‐Nt	hα‐DG‐Nt	Res	mα‐DG‐Nt	hα‐DG‐Nt
92	Asp	Glu	81	Ser	Thr	212	Asn	His	275	Arg	His
136	Ala	Thr	112	Pro	Ser	215	Gln	Arg	279	Thr	Ala
145	Val	Ile	113	His	Gln						
			116	Ile	Thr						

**Figure 3:**
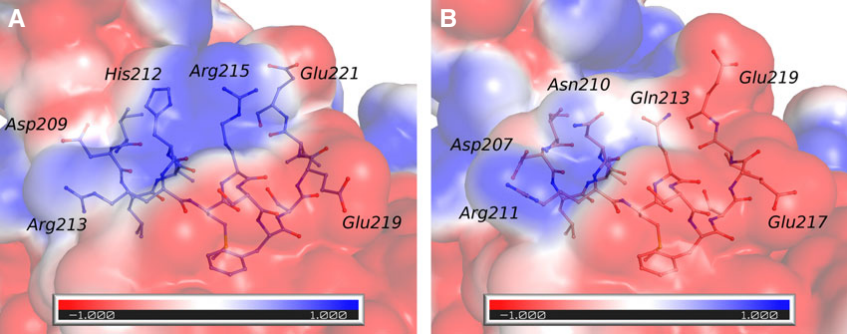
Electrostatic potential maps of mα‐DG‐Nt and hα‐DG‐Nt. The electrostatic potential (kbT/ec) is mapped on the human and murine α‐DG‐Nt‐accessible surfaces. Molecular models are represented as ribbons with selected residues depicted as stick‐and‐ball. (A) hα‐DG‐Nt, 208–221 stretch. (B) mα‐DG‐Nt, 206–219 stretch.

While the global rmsd is quite low, small but significant deviations between superposed *C*_α_ *s* (around 0.7–1.2 Å against average values of ~ 0.1–0.3 Å and a maximum‐likelihood error estimate of 0.233 Å) are observed in the zones encompassing residues 111–114, 134–145, and 155–162. The zone defined by residues 134–145, which includes N‐ and C‐terminal regions of β‐strands F and G and the turn connecting them 18, does not show any remarkable structural variation and the *C*_α_ *s* deviations above the average are probably due to the intrinsic dynamics of the turn connecting the two strands. Apart from the flexible linker (residues 161–181) connecting the Ig‐like and the S6 domains, the zone encompassing residues 111–114 shows the highest deviations between superposed *C*_α_ *s* (Fig. S2A), which is probably due to the very different nature of the residues occupying the same topological position in the two proteins (Pro110 in mα‐DG‐Nt and Ser112 in hα‐DG‐Nt). It is well known that prolines reduce the conformational freedom of the proteins backbone 25, and it is likely that its substitution in the corresponding position of hα‐DG‐Nt with Ser112 may affect the local main chain conformation. Indeed, in hα‐DG‐Nt, residues 112–114 assume a helix‐3_10_ conformation, instead of the turn observed in mα‐DG‐Nt. We do not observe any relevant backbone deviation between mα‐DG‐Nt and hα‐DG‐Nt for the residues being part of patches P3 and P4. Nonetheless, the interacting network of residues spatially closer to His212 and Arg215 (patch P3) is different from that observed in the corresponding region of mα‐DG‐Nt (Fig. 3A–B). This discrepancy is likely due to the different chemical nature of the positively charged His and Arg amino acids (hα‐DG‐Nt) with respect to the polar Asn and Gln residues (mα‐DG‐Nt). This notion is further supported by the comparison of the electrostatic potentials of mα‐DG‐Nt and hα‐DG‐Nt, which are locally different around residues 212 and 215 (Fig. 3A–B), while overall being quite similar (Fig. S1A‐B).

The hα‐DG‐Nt and mα‐DG‐Nt crystal structures display the most significant structural differences in the flexible linker (see Fig. S2A,B) connecting the Ig‐like and S6 domains. According to the hα‐DG‐Nt refined model, the conformation of the N‐terminal part of the linker, the only one reliably modeled in both structures, differs from that observed in mα‐DG‐Nt crystal structure. This finding is not surprising, being the linker very flexible 20, probably playing a pivotal role in α‐DG structural plasticity as discussed in the next paragraphs. As mentioned above, the linker is also one of the α‐DG zones with the highest sequence variability among different species (see 26 and Table S1). Besides, the presence of Leu164 in hα‐DG‐Nt (not modeled in the hα‐DG‐Nt crystallographic structure) instead of Pro162 in the corresponding position of mα‐DG‐Nt may influence hα‐DG‐Nt conformational variability with respect to mα‐DG‐Nt. Indeed, residues 159–161 assume a helix‐3_10_ conformation in hα‐DG‐Nt, whereas they display a turn/coil conformation in mα‐DG‐Nt.

### Association state and overall parameters of the N‐terminal domains of murine and human α‐dystroglycan in solution

Small‐angle X‐ray scattering experiments were performed to compare the conformations in solution of mα‐DG‐Nt and hα‐DG‐Nt.

The protein solutions were analyzed at different concentrations (Table 3), and in both cases, no systematic changes with the solute concentration could be observed, although the murine protein showed a certain degree of aggregation, likely due to the higher concentration of the stock solution.

**Table 3:** Overall parameters calculated from SAXS data analysis

Samples	Concentration (mg·mL^−1^)	*R*_g_ (Å)	*D*_max_ (Å)	*V*_p_ (Å^3^)	MM_exp_ (kDa)	χ_crystal_	χ_CORAL_	χ_*ab initio*_	χ_EOM_
mα‐DG‐Nt	0.22–3.38	25.2 ± 0.04	90 ± 3	44 000 ± 2000	25.1 ± 3.0	2.87	0.84	0.81	0.63
hα‐DG‐Nt	0.24–4.2	25.1 ± 0.04	90 ± 3	44 000 ± 2000	28.8 ± 3.0	2.25	1.17	1.14	1.06

Notations: MMexp, experimental molecular mass of the solute; χ_crystal_, χ_CORAL_, χ_*ab initio*_, and χ_EOM_, discrepancy (chi‐square value) for the fit from the crystallographic structures with the missing regions reconstructed by CORAL keeping fixed the two domains, from rigid body modeling using CORAL, from *ab initio* modeling using DAMMIN, and from EOM, respectively.

**Figure 4:**
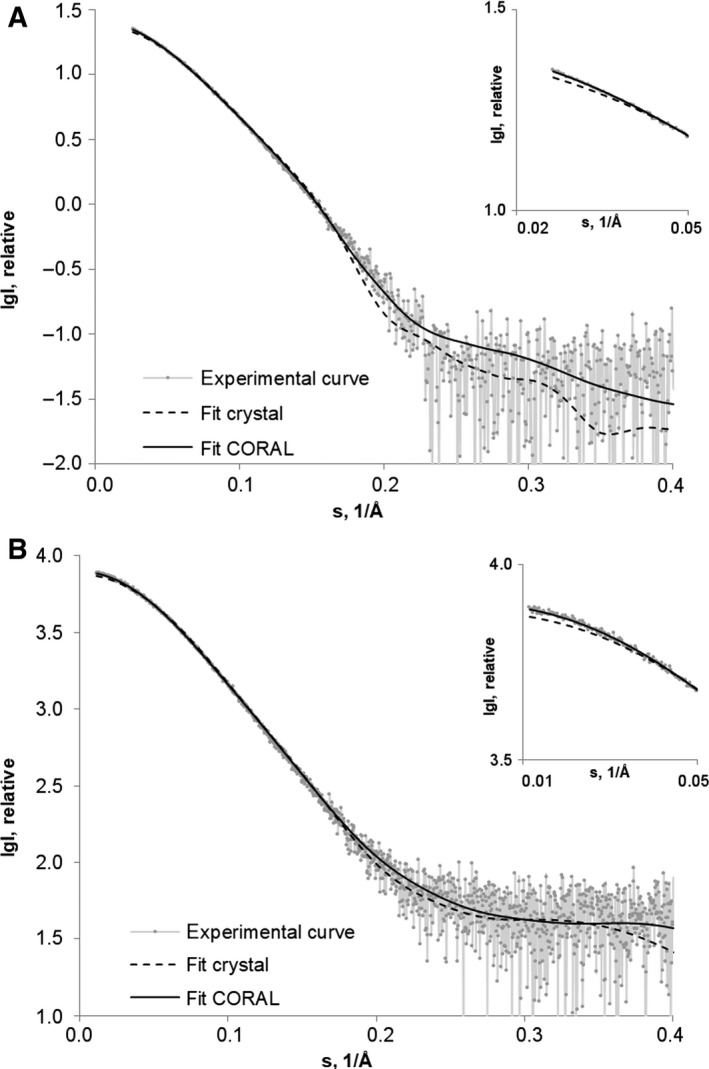
Experimental X‐ray scattering data and obtained fits for (A) mα‐DG‐Nt and (B) hα‐DG‐Nt. Experimental SAXS patterns, scattering calculated from the crystallographic models (‘fit crystal’, continuous line), scattering calculated from rigid body models obtained by CORAL (‘fit CORAL’, dashed line). The plots display the logarithm of the scattering intensity as a function of momentum transfer. The zoomed regions of these graphs at low angles are presented in the insert.

**Figure 5:**
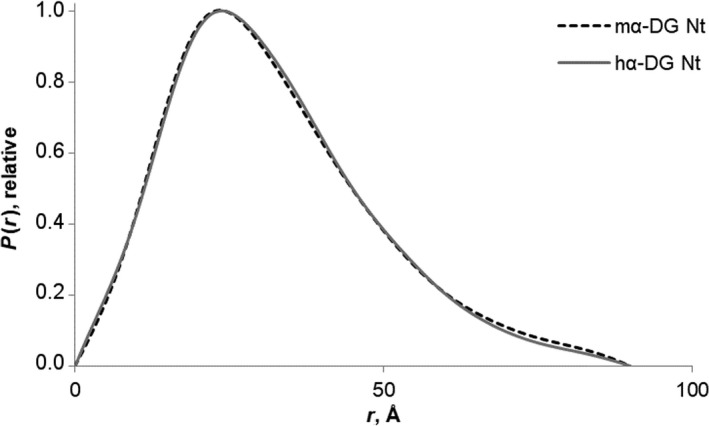
Distance distribution functions for mα‐DG‐Nt and hα‐DG‐Nt. Overlaid of the *p*(*r*) distance distribution functions calculated from the experimental SAXS data of mα‐DG‐Nt (black dotted line) and hα‐DG‐Nt (gray solid line).

The experimental SAXS curves, obtained at the highest concentration of mα‐DG‐Nt and hα‐DG‐Nt, are displayed in Fig. 4A,B, respectively, and the computed *p*(*r*) distance distribution functions are displayed in Fig. 5; the overall parameters extracted from the SAXS data are summarized in Table 3 (additional details on SAXS structural parameters are reported in Table S2).

The molecular mass (MM) of the proteins, estimated from the relative forward scattering intensities (*s* = 0, with *s* the scattering vector), suggests that both proteins are monomeric in solution at all conditions tested and is in good agreement with the value estimated from the primary sequences (around 28.5 kDa). This is further corroborated by excluded volume of the hydrated protein molecules (*V*_p_), consistent with the empirical finding for globular proteins that the hydrated volume expressed in nm^3^ should numerically be about twice the MM in kDa. The experimental radius of gyration (*R*_g)_ and maximum size (*D*_max_; Table 3) point to an elongated shape of the proteins, and the two *p*(*r*) functions that nicely overlap (Fig. 5) display an asymmetric tail, typical of elongated particles.

It is interesting to note that in both cases, the scattering curves computed by the crysol program 27 based on the crystallographic models (PDB ID 1U2C and 5LLK for mα‐DG‐Nt and hα‐DG‐Nt, respectively) give a poor fit to the respective experimental data (data not shown). Even upon reconstruction of the missing regions (around 10 amino acids at both N‐terminal and C‐terminal regions and the missing linker between the two domains that are kept fixed) using CORAL 28, the fit is not improved (χ_crystal_ in Table 3 and ‘fit crystal’ in Fig. 4 with the zoomed portions at low angles in the inserts). It can be thus concluded that both murine and human α‐DG‐Nt are monomeric in solution, even at relatively high concentrations, but they show a significantly more extended conformation than in the crystallographic models.

### Molecular shape reconstruction of the N‐terminal domains of murine and human α‐dystroglycan in solution

Two different strategies have been employed to reconstruct the macromolecular shapes of the two proteins in solution.

**Figure 6:**
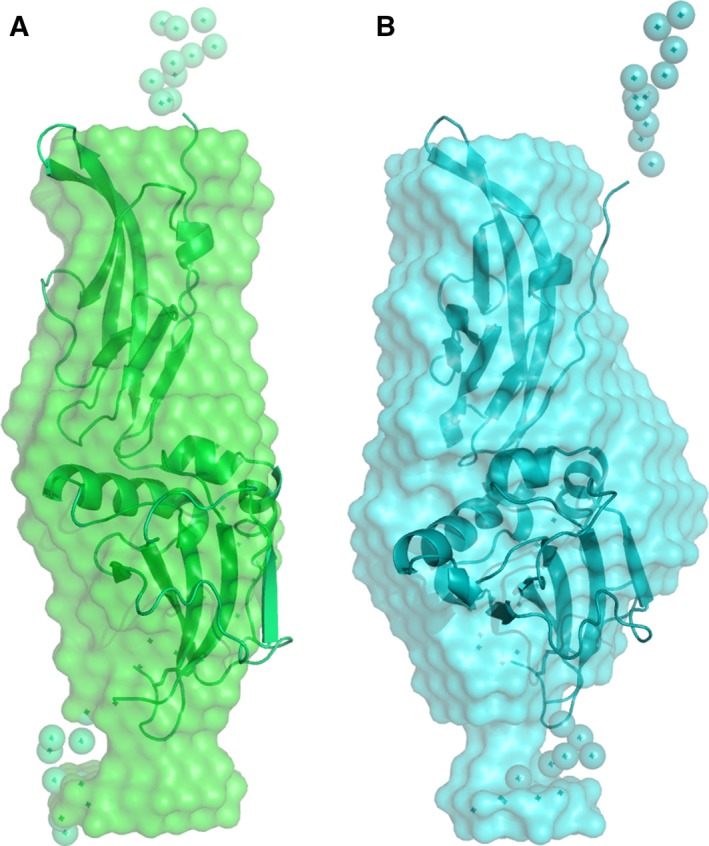
Structural models of mα‐DG‐Nt (A) and hα‐DG‐Nt (B). Averaged and filtered *ab initio* beads models as obtained by DAMMIN (green and cyan semitransparent surfaces) superimposed on the typical CORAL models (green and cyan cartoon representations for the folded Ig‐like and S6 domains, spheres for the restored missing fragments).

At first, low‐resolution three‐dimensional models of mα‐DG‐Nt and hα‐DG‐Nt were reconstructed from the experimental X‐ray scattering data using the *ab initio* modeling program DAMMIN 29 (Fig. 6), with all models providing an excellent fit to the experimental data (Table 3, χ_*ab initio*_). The final DAMMIN models are the result of analyzing and averaging 10 independent solutions. The normalized spatial discrepancy (NSD) value, which describes the similarity between the different models produced by the program 30, is low for both the murine and human models (0.478 ± 0.029 and 0.547 ± 0.014, respectively), indicating that the multiple solutions built by the program are very similar to each other. The visible similarity in the shapes of the two models suggests that at low resolution, no differences could be detected.

A second approach to molecular shape reconstruction consisted in a rigid body modeling of the two proteins from the scattering data conducted by using the program CORAL 28. High‐resolution models of individual Ig‐like and S6 domains in the corresponding crystal structures were combined with different conformations of flexible dummy residue linkers. The relative orientations of the two domains and the reconstruction of the flexible missing loops linking the Ig‐like and the S6 domains were optimized. The CORAL models nicely overlay with the scattering curves (χ_CORAL_ in Table 3 and ‘fit CORAL’ in Fig. 4 with the zoomed portions at low angles in the inserts) and well superpose into the SAXS envelopes of the *ab initio* models (Fig. 6). It is interesting to notice that, probably due to packing stabilizing interactions of the crystal lattice, the two crystallographic structures are more compact than the respective conformations in solution, whose more elongated shapes are most evident in the asymmetric tail at the higher r of their *p*(*r*) distributions (Fig. 5). As a quantitative measure of structure compactness, the distances between centers of masses of the Ig‐like and S6 domains in the crystallographic models (29.7 and 29.9 Å in the human and murine models, respectively) have been compared to those of the CORAL models (33.6 and 34.4 Å in the human and murine models, respectively), confirming the existence of more extended conformations in solution. The most straightforward explanation is that the solution structures of the N‐terminal regions of murine and human α‐DG display a conformation that is more flexible than the one inferred from their crystal structures.

### Interdomain flexibility of the N‐terminal domains of murine and human α‐dystroglycan in solution

More extended mα‐DG‐Nt and hα‐DG‐Nt solution structures are in agreement with the evidence of a disordered region linking the Ig‐like and S6 domains, as suggested by the crystallographic analysis. Indeed, linker flexibility could allow variability in the relative orientation of the individual domains, resulting in structural plasticity.

**Figure 7:**
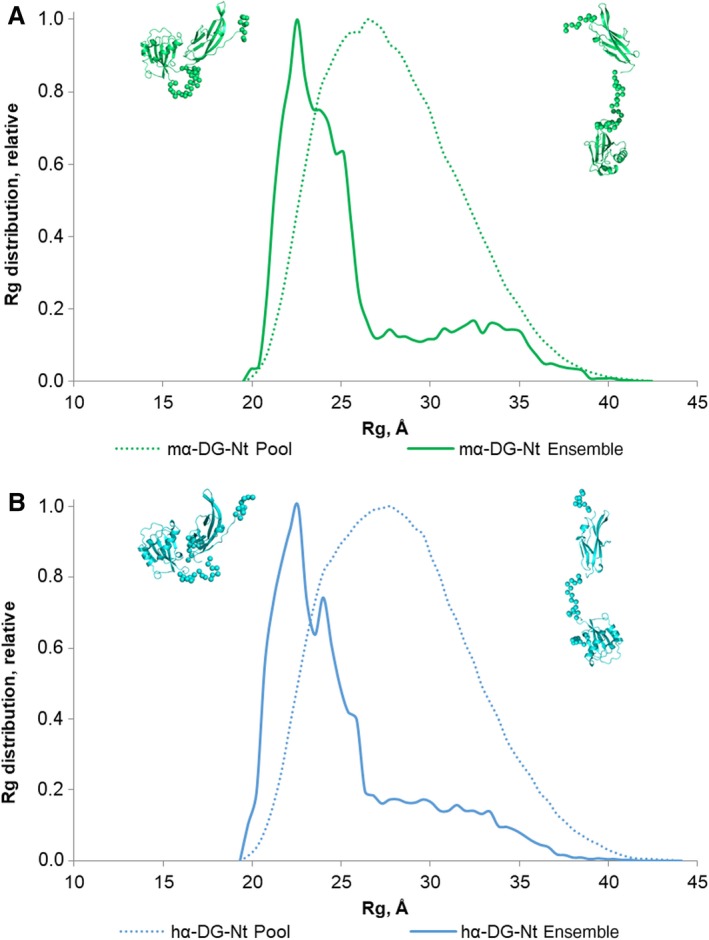
*R*_g_ distributions for the EOM models of mα‐DG‐Nt (A) and hα‐DG‐Nt (B). The distributions for the initial random pools of models are shown as green and blue dot lines; green and blue solid lines correspond to the selected ensembles. The representative conformations are shown near the distributions: compact conformation on the left and extended conformation on the right of the respective distributions. Folded domains are depicted as cartoons (mα‐DG‐Nt in green and hα‐DG‐Nt in blue); linkers and the N‐terminal and C‐terminal reconstructed regions are represented by spheres.

An analysis of the interdomain flexibility and size distribution of possible multiple configurations in solution was conducted by using the ensemble optimization method (EOM) 31, obtaining typical optimized ensembles that fit well the measured scattering data (Table 3). The EOM analyses of the murine and hα‐DG‐Nt are presented in Fig. 7 as a size distribution, plotting the *R*_g_ of the structures forming the random pool and the selected ensembles against their relative frequencies.

The *R*_g_ distributions of these ensembles (Fig. 7, solid lines) are very similar to each other and nearly as broad as the distribution of randomly generated models (Fig. 7, dashed lines), supporting the hypothesis of a certain degree of interdomain flexibility. Moreover, these *R*_g_ distributions are both characterized by a bimodal profile. Indeed, the predominant fractions (around 60%) represent relatively compact models with *R*_g_ of about 21–25 Å, while a very small fraction (around 15%) of models with *R*_g_ of about 30–35 Å is due to more elongated configurations: two molecular structures representative of different conformations are shown in Fig. 7 (compact conformations on the left and extended conformations on the right). This interdomain flexibility may confer structural plasticity, which in turn may represent the molecular basis regulating α‐DG maturation and/or modulating the interactions with other physiological binding partners.

## Discussion

The aim of this study was to investigate the comprehensive structure of α‐DG‐Nt in solution. By exploring for the first time the conformational landscape of human and mouse α‐DG‐Nts in solution by means of SAXS experiments, we disclosed unexpected shared features of α‐DG‐Nt that may help to elucidate the molecular basis of the physiological and pathological functions of α‐DG. Besides, the analysis of the SAXS and crystallographic data of the two orthologs validates previous results and the structure of mα‐DG‐Nt as a fully descriptive model for hα‐DG‐Nt. This notion is further supported by the comparison of the conformational stability of the two orthologs as assessed by biochemical and biophysical experiments.

The hα‐DG‐Nt crystal structure displays the same fold of mα‐DG‐Nt, with few structural differences. It is interesting to note that when mapping the not conserved amino acids on the high‐resolution 3D structure, they cluster around four distinct patches, which span along only one edge of the α‐DG‐Nt longest axis (Figs 2 and S4). Such clustering might have a biological significance; that is, these patches might represent ‘hotspots’ for transient species‐specific protein–ligand interactions. On the other hand, the conserved surface involving especially the S6 domain (Fig. S1) may be of functional relevance for LARGE recognition along the α‐DG maturation pathway. Moreover, it is interesting to note that the patch involving residues 112 and 113 in hα‐DG‐Nt just follows Asp111, whose mutation to Asn has been related to pathological α‐DG hypoglycosylation 8, suggesting that this residue is involved in the complex mechanism leading to the mature, fully glycosylated α‐DG.

In order to explore the molecular structure of the α‐DG‐Nt in solution, we have undertaken a SAXS study. SAXS is the technique of election for low‐resolution structural studies in solution, especially valuable for flexible systems, whose conformational variability description is precluded to crystallography 32. While SAXS cannot infer the molecular structure at the atomic level like X‐ray crystallography can mostly do, it offers the unique opportunity to obtain, albeit at lower resolution, a structural model in solution, free of the packing forces that may instead influence a crystallographic model 33. Indeed, while packing effects are not expected to affect the compact folds of the single domains, they could in this case have an impact on the relative orientation of the Ig‐like and the S6 domains. Mutual domains orientation may be assisted by the flexible 20‐residue‐long linker connecting them, which may influence the overall conformation of the α‐DG‐Nt. Combined with the crystallographic models of murine and hα‐DG‐Nt s, SAXS analysis may provide a reliable low‐resolution model of α‐DG at near‐physiological conditions 34.

The SAXS study presented here points to structural models significantly different from those observed in the murine and hα‐DG‐Nt crystal structures. According to their solution structures obtained by SAXS data analysis exploiting the respective crystal structures, mα‐DG‐Nt and hα‐DG‐Nt both assume a remarkably less compact structure in solution than that observed in the crystal structures. The comparison of mα‐DG‐Nt and hα‐DG‐Nt *p*(*r*) distributions pinpoints to a more extended conformation, a feature that is notably similar for the two orthologs in solution (Fig. 5). Furthermore, the mα‐DG‐Nt and hα‐DG‐Nt SAXS models nicely fit on the low‐resolution molecular envelopes obtained by DAMMIN *ab initio* method. Employing the program CORAL 28, apart from rigid body fitting of the Ig‐like and S6 domains, we have been able to reconstruct the missing parts of both mα‐DG‐Nt and hα‐DG‐Nt. While the N‐ and C‐terminal zones of the Ig‐like and S6 domains, respectively, assume extended conformations, the linker connecting the two subdomains displays a more compact structure, rather similar in the two proteins despite important differences in their amino acid sequences. It is interesting to note that by comparing the crystal structures with the CORAL models, the S6 domain appears to be rotated by about 90° to each other around the Ig‐like domain. Even if this observation might suggest some functional implications for this assembly, it must be taken into account that the CORAL models have some intrinsic limitations due to the assumption of the models to be rigid. Indeed to overcome this bias and to gain further information on the conformational variability in mα‐DG‐Nt and hα‐DG‐Nt in solution, the interdomain flexibility has been assessed by using the EOM method 31. According to our analysis, murine and hα‐DG‐Nt share a common behavior in solution, as shown by the similarity in their bimodal *R*_g_ distribution curves, with comparable maxima and slightly different frequencies. Indeed, both mα‐DG‐Nt and hα‐DG‐Nt in solution are partitioned among few principal populations, which differ in their compactness (Fig. 7). More extended conformations seem to be present, but their relative abundance is low when compared with the most frequent. The common conformational characteristics of murine and hα‐DG‐Nt suggest that in solution α‐DG may have a more complex behavior than expected only on the basis of its crystal structures.

According to the present SAXS study, the structural plasticity of the α‐DG‐Nt seems to be a general property of this protein, as mα‐DG‐Nt and human α‐DG‐Nt show common structural features. Such unexpected conformational variability of the α‐DG‐Nt is of great interest and it may play a functional role in its ability to interact with different partners, either inside the cell along its maturation pathway or at the level of the ECM, or in both. It has been proposed that α‐DG‐Nt can assist the bifunctional glycosyltransferase LARGE in its complex enzymatic actions, a function that may require α‐DG‐Nt to assume different, functionally relevant conformational states. It is also tempting to speculate that the functional flexibility of the α‐DG‐Nt was positively selected as it conferred a strong advantage for the multistep maturation pathway. Along this pathway, concerted connections must be established between a plethora of glycosyltransferases and regulatory enzymes that extensively decorate the mucin‐like region within the Golgi lumen 35. Further biochemical and structural work is warranted in order to assess the possibility of direct interactions of α‐DG‐Nt with some of these enzymes. Furthermore, the conformational variability in α‐DG highlights the consolidated notion that α‐DG displays a distinct structural modularity, in line with the recent analysis of α‐DG conserved domain organization in metazoan 36. The autonomous folding modular nature of the entire α‐DG‐Nt 26 prompted its possible use as a serum biomarker in DMD patients 37 or also in human uterine fluid to determine uterine receptivity 38.

In line with these results, molecular plasticity of α‐DG in solution should be considered and investigated to enhance our understanding of the molecular basis of the physiological and pathological role of a central component of the dystrophin–glycoprotein complex.

## Materials and methods

### Cloning, expression, and purification

As previously reported for the murine α‐DG N‐terminal region, mα‐DG‐Nt(50–313)R168H 18, we have cloned the DNA fragment encoding for its human counterpart, hα‐DG‐Nt(52–315), into the bacterial vector pHis‐Trx, for the expression of the protein as a thioredoxin fusion product, with an N‐terminal His_6_ tag and a thrombin cleavage site. We also introduced within hα‐DG‐Nt(52–315) the additional mutation R170H, in order to make the protein more proteolytically resistant 22. The recombinant hα‐DG‐Nt(52–315)R170H was obtained as previously reported 20. Namely, it was expressed as fusion protein in *Escherichia coli* BL21(DE3) Codon Plus RIL, purified by nickel affinity chromatography, cleaved by thrombin, and further purified by anion‐exchange and gel filtration chromatography.

### Differential scanning fluorimetry (DSF)

Differential scanning fluorimetry experiments were carried out on a CFX96 Touch Real‐time PCR instrument (Bio‐Rad, Hercules, CA, USA). Measurements were taken using an excitation wavelength of 470–505 nm and an emission wavelength of 540–700 nm. Data were acquired using a temperature gradient from 20 to 90 °C in 0.2 °C·min^−1^ increments. The samples contained 0.5 mg·mL^−1^ murine and hα‐DG‐Nt proteins in 20 mm Tris, 150 mm NaCl pH 7.5, and 90× SYPRO Orange (Sigma‐Aldrich, St. Louis, MO, USA) in a total volume of 25 μL. The melting curves represent the fluorescence increase arising from the association of SYPRO Orange with exposed hydrophobic residues as the protein unfolds with increasing temperature 33. Experiments were performed in triplicate. Fluorescence data were analyzed and the *T*_m_, represented by the inflection points of the transition curves, were calculated using the Boltzmann sigmoid fit 22.

### Limited proteolysis

mα‐DG‐Nt and hα‐DG‐Nt were subjected to limited proteolysis at 37 °C at a final concentration of 30 μm in 25 mm Tris pH 7.5, 150 mm NaCl buffer. A panel of proteases from Proti‐Ace kits (Hampton Research, Aliso Viejo, CA, USA), that is, bromelain, proteinase K, subtilisin, thermolysin, trypsin, endoproteinase Glu‐C, and clostripain (endoproteinase Arg‐C), were tested at a final concentration of 2 μg·mL^−1^. The reactions were stopped after 1, 5, 10, 20, 40, and 60 min by adding SDS sample buffer to aliquots of the reaction mixtures. The samples were analyzed by performing 15% SDS/PAGE and Coomassie staining.

### Crystallization, data collection, structure solution, and refinement

Attempts to grow crystals of hα‐DG‐Nt by using the hanging‐drop vapor diffusion method from conditions similar to those previously reported for both wild‐type mα‐DG‐Nt and its mutant T190M 18, 20 were not successful. While exploring new crystal growth conditions by using commercial high‐throughput screening kits (100 + 100 nL of protein and precipitant solution at both 277 and 297 K), we also attempted cross‐seeding methods starting from already‐grown crystals of mα‐DG‐Nt mutant. While the screenings did not reveal new crystallization conditions, cross‐seeding, based on well‐established protocols 39, 40, resulted in the growth of well‐shaped crystals. Precipitant conditions (0.6–1.4 m sodium citrate buffer) and pH (6.8–7.2) were screened by mixing 1 μL of protein solution (10 mg·mL^−1^ hα‐DG‐Nt in 150 mm NaCl, 25 mm Tris, pH 7.5) with 1 μL of precipitant solution; the drops were equilibrated at 277 K for 3–6 days before seeding. Fully grown crystals were obtained in 2 weeks after seeding in the optimized growth conditions (0.8 m sodium citrate buffer, pH 7.2). Both streak‐seeding and microseeding methods were attempted with seeds stock prepared following the manufacturer's protocol (Hampton Research, HR2‐320 User Guide). The best‐shaped crystals were obtained by using the streak‐seeding method. Repeated streak seeding (two to three times), following the same protocol but at optimal precipitant and pH conditions, increased crystal dimensions and improved their diffraction quality.

Data collections were carried out at the XRD1 beamline at ELETTRA (Trieste, Italy) 41, 42 using a Pilatus‐2M (Dectris Ltd., Baden, Switzerland) detector and the wavelength of 1.00 Å. Data collection was carried out at 100 K after having quickly dipped the crystals into a cryoprotectant solution (25% ethylene glycol added to the precipitant solution) and frozen in liquid nitrogen. Indexing, integration, and data reduction of the diffraction data were carried out by the xds program 43. Data reduction statistics of the hα‐DG‐Nt dataset are reported in Table 4.

**Table 4:** X‐ray diffraction: data collection and model refinement statistics

Data collection	
PDB_ID	5LLK
Space group	H3
Unit cell parameters (Å); *a, c*	71.8, 144.0
Molecules per asymmetric unit	1
Wavelength (Å)	1.000
Resolution (Å)	48.0–1.80 (1.85–1.80)^a^
Total observations	175 979 (12 160)
Unique reflections	25 220 (1766)
*R*_merge_ b	0.046 (1.070)
*R*_meas_ c	0.049 (1.164)
CC_1/2_	1.000 (0.868)
<*I*/σ (*I*)>	21.60 (1.61)
Completeness (%)	99.0 (94.5)
Redundancy	7.0 (6.9)
Refinement	
Resolution (Å)	36–1.80 (1.864–1.80)^a^
Number of reflections (work set/test set)	23 910/2419
*R*_work_ d	0.1641 (0.3862)
*R*‐_free_ e	0.1947 (0.3907)
Number of non‐H atoms	
Protein	1702
Waters	129
Organic (ethylene glycol)	8
Average isotropic B‐factors (Å^2^)	
Protein	44.0
Ligand	53.6
Solvents	44.9
Rmsd from ideality	
Bond length (Å)	0.01
Angle (deg)	1.01
Ramachandran plot	
Favored regions (%)	99.0
Allowed regions (%)	1.0
Disallowed regions (%)	0.0

a Values in parentheses are given for the highest resolution shell.

b *R*_merge_ = ∑_*hkl*_∑_*j*_│*I*_*hkl,j*_ − <*I*_*hkl*_>│/∑_*hkl*_∑_*j*_ *I*_*hkl,j*_.

c *R*_meas_ = ∑_*hkl*_ [*n*/(*n* − 1)]^1/2^∑_*j*_│*I*_*hkl,j*_ − <*I*_*hkl*_>│/∑_*hkl*_∑_*j*_ *I*_*hkl,j*_

d *R*_work_ = ∑_work set_│*F*_obs_ − *F*_cal_│/∑_work set_ *F*_obs_.

e *R*‐_free_ = ∑_test set_ |*F*_obs_ − *F*_cal_|/∑_test set_ *F*_obs_.

The structure solution of hα‐DG‐Nt was obtained by Patterson search methods, using the phaser software, implemented in the PHENIX crystallographic package 44. The crystal structure of WT mα‐DG‐Nt (PDB ID: 1U2C 18) was used as a search template. Rigid body refinement was initially carried out, followed by a simulated annealing step. The following cycles of the crystallographic refinement included positional refinement and translation‐libration‐screw (TLS) model parameterization before the individual B‐factors refinement. All the refinement cycles were carried out by using phenix.refine 45 and were alternated with the manual rebuilding of the structure by using the coot software 46. Solvent molecules were included in the final model by using the automatic search protocol available in phenix.refine and manually checked before being included in the model. Occupation of residues Asp160, His161, Ser162, Ala184, and Asp185 that display a poor electron density and a high B_iso_ was also refined. Protein stereochemistry was monitored throughout the refinement process and during manual rebuilding with MolProbity 47. Statistics of the crystallographic refinement are reported in Table 4. Coordinates and structure factors have been deposited in the PDB, with accession number 5LLK. Molecular diagrams were created with pymol 48 and the stride web server 49 has been used for hα‐DG‐Nt secondary structure assignments. Protein structures superposition and rmsd estimation were carried out by ProFit (Martin, A.C.R., http://www.bioinf.org.uk/software/profit).

### Small‐angle X‐ray scattering (SAXS)

Small‐angle X‐ray scattering data for mα‐DG‐Nt were collected on the BM29 beamline 50 at the European Synchrotron Radiation Facility (ESRF, Grenoble, France) as 10 × 1 s exposure time using a Pilatus 1M detector, sample detector distance of 2.87 m and wavelength of 0.99 Å. SAXS measurements for hα‐DG‐Nt were taken on the P12 beamline EMBL SAXS‐WAXS at PETRAIII/DESY (Hamburg, Germany) 51 as 20 × 0.05 s exposures time using a Pilatus 2M detector, sample detector distance of 3.00 m and wavelength of 1.24 Å. Scattering profiles for the collected frames were compared to detect radiation damage.

Measurements were taken at six different concentrations (the ranges are reported in Table 3; additional details on SAXS structural parameters are reported in Table S2) in 20 mm Tris, 150 mm NaCl, pH 7.5.

After normalization to the intensity of the transmitted beam, frames were merged for each sample. Subtraction of the buffer's contribution to the scattering and further processing steps were performed using primus 52 from the atsas 2.6.0 program package 28. The forward scattering *I*(0) and the *R*_g_ were evaluated using the Guinier approximation 53, assuming that at very small angles (*s* < 1.3/*R*_g_), the intensity is represented as:

$$I\left(s\right)=I\left(0\right)\;\times \;e^{\frac{-{\left(s\times R_{g}\right)}^{2}}{3}}.$$

Pair distance distribution functions of the particles *p*(*r*) and the maximum sizes *D*_max_ were computed using GNOM 54. MM was estimated by comparison of the calculated forward scattering *I*(0) of the samples with that of the standard solution of bovine serum albumin (MM 66 kDa). *V*_p_ was calculated using the Porod approximation 55:

$$V_{p}=\frac{2\pi^{2}I\left(0\right)}{\int I_{\exp}\left(s\right)s^{2}ds}.$$

The program DAMMIN 29 was employed to construct low‐resolution *ab initio* beads models of murine and hα‐DG‐Nt that best fit the scattering data. It employs a simulated annealing procedure to build a compact bead configuration inside a sphere with the diameter *D*_max_ that fits the experimental data *I*_exp_(*s*) to minimize the discrepancy:

$$\chi^{2}=\frac{1}{\left(N-1\right)}\sum_{j}{\left[\frac{I_{\exp}\left(s_{j}\right)-cI_{\mathrm{calc}}\left(s_{j}\right)}{\sigma (s_{j})}\right]}^{2}.$$

Ten independent DAMMIN runs were performed for each scattering profile in the ‘slow’ mode, using default parameters and no symmetry assumptions (P1 symmetry). The models resulting from independent runs were superimposed using the program SUPCOMB 30, and aligned models were averaged using DAMAVER 56 to generate a consensus three‐dimensional shape.

A simulated annealing protocol implemented in CORAL 28 was employed to find the optimal positions and orientations of the available high‐resolution models of the Ig‐like and S6 domains of murine and human α‐DG‐Nt. In addition, the program also generated approximate clash‐free conformations of the missing portions of polypeptide chain (around 10 amino acids at both the N‐terminal and C‐terminal regions and the missing linker between the Ig‐like and S6 domains). The model fit of the X‐ray crystal structures (PDB ID: 1U2C for mα‐DG‐NT and 5LLK for hα‐DG‐Nt) against the SAXS data was calculated using crysol 27.

Analysis of the interdomain flexibility and size distribution of possible conformers, consistent with the measured scattering data for murine and human α‐DG‐Nt, was conducted using the EOM 31. This method selects an ensemble of possible conformations from a pool of 10 000 randomly generated models constructed from rigid domains linked by randomly generated flexible linkers. The program crysol is used to calculate the theoretical scattering profiles of these models, and a genetic algorithm, GAJOE, is used to select an ensemble of conformations, whose combined scattering profiles best fit the experimental data. The crystal structures of the Ig‐like and S6 domains of murine and human α‐DG‐Nt were used as rigid bodies for the analysis of the scattering data, employing ensemble optimization. Linkers between the domains and missing N‐terminal and C‐terminal stretches were represented as a flexible chain of dummy residues.

### Sequence alignment

Murine and human α‐DG sequences were aligned in muscle 3.8 57 available at EMBL/EBI (http://www.ebi.ac.uk/Tools/msa).

## Data accessibility

Structural data are available in PDB database under the accession number 5LLK.

## Author contributions

SC, MB, AB, and AC conceived and designed the experiments. SC, MB, MGB, FS, PVK, and AC performed the experiments. SC, MB, MGB, FS, PVK, AB, and AC analyzed the data. SC, MB, AB, and AC wrote the manuscript. All the authors have approved the manuscript.

## Supporting information


**Table S1**. Multiple alignment of selected mammalian sequences of the N‐terminal region of α‐DG.
**Table S2**. SAXS structural parameters.
**Figure S1**. Electrostatic potential maps of ma‐DG‐Nt and ha‐DG‐Nt.
**Figure S2**. Superimposition of the ha‐DG‐Nt and ma‐DG‐Nt selected regions.
**Figure S3**. Amino acid differences between mouse and human α‐DG‐Nt mapped onto the hα‐DG‐Nt accessible surface.
**Figure S4**. Amino acid differences mapped onto the hα‐DG‐Nt accessible surface.
