# Supplementary material for: Structural flexibility of human α‐dystroglycan
Source: FEBS Open Bio. 2017 Jul 17;7(8):1064–77. doi: 10.1002/2211-5463.12259 (PMC5537065; doi:10.1002/2211-5463.12259)
Supplement: Supplementary file 1 — Table S1. Multiple alignment of selected mammalian sequences of the N‐terminal region of α‐DG. Table S2. SAXS structural parameters. Figure S1. Electrostatic potential maps of ma‐DG‐Nt and ha‐DG‐Nt. Figure S2. Superimposition of the ha‐DG‐Nt and ma‐DG‐Nt selected regions. Figure S3. Amino acid differences between mouse and human α‐DG‐Nt mapped onto the hα‐DG‐Nt accessible surface. Figure S4. Amino acid differences mapped onto the hα‐DG‐Nt accessible surface. [file FEB4-7-1064-s001.pdf]

SUPPLEMENTARY MATERIAL FOR:

Structural flexibility of human α-dystroglycan.

Sonia Covaceuszach, Manuela Bozzi, Maria Giulia Bigotti, Francesca Sciandra, Petr Valeryevich Konarev, Andrea Brancaccio, Alberto Cassetta

Contents:

Table S1: SAXS structural parameters.

Table S2: Multiple alignment of selected mammalian sequences of the N-terminal region of α-DG.

Figure S1: Electrostatic potential maps of mα-DG-Nt and hα-DG-Nt.

Figure S2: Superimposition of the hα-DG-Nt and mα-DG-Nt selected regions.

Figure S3: Amino acid differences between mouse and human α-DG-Nt mapped onto the hα-DG-Nt accessible surface.

Figure S5: Amino acid differences mapped onto the hα-DG-Nt accessible surface.

**Data collection parameters**	hα-DG-Nt	mα-DG-Nt
Instrument	P12 (PETRA III)	BM29 (ESRF)
Beam size (mm^2)	0.2 x 0.12	0.5x0.5
Wavelength (Å)	1.24	0.99
q range ($Å^{-1}$)	0.003–0.445	0.0025–0.6
Concentration range (mg / mL)	0.24-4.20	0.22–3.38
Temperature (K)	283	283
**Structural parameters**		
I(0) (A.U.) [from p(r)]	7944±20	26.0±0.06
R_g (Å) [from p(r)]	25.2±0.04	25.7±0.04
I(0) (A.U.) [from Guinier]	7972±20	25.9±0.06
R_g (Å) [from Guinier)]	25.1±0.04	25.2±0.04
D_{max} (Å)	90±3	90±3
Porod volume estimate ($Å^3$)	44000±2000	44000±2000
**Molecular mass determination (Dalton)**		
Molecular mass MM [from I(0)]	28800±3000	25100±3000
Molecular mass MM [from Porod volume]	26000±3000	26000±3000
Calculated monomeric M_r from sequence	28500	28500
**Software employed**		
Primary data reduction	PRIMUS	PRIMUS
Data processing	GNOM	GNOM
Ab initio analysis	DAMMIN	DAMMIN
Validation and averaging	DAMAVER	DAMAVER
Rigid body modeling	CORAL/EOM	CORAL/EOM
Computation of model intensities	CRYSOL	CRYSOL
Three-dimensional graphic representations	PYMOL	PYMOL

Table ~~S1~~. SAXS structural parameters: radius of gyration (R_g), maximum dimension (D_{max}), Porod volume ($Å^3$), and MM (Dalton). D_{max} was obtained from the p(r) distribution using GNOM; I(0) (scattering intensity) was obtained from the scattering data by the Guinier analysis. Molecular mass (MM) was estimated from comparison with I(0) intensity of the standard BSA sample.

```
Mm              SVLSDFQEAVPTVVGIPDGTAVVGRSFRVSIPTDLIASSGEIIKVSAAGKEALPSWLHWD
Rn              SVLSDFQEAVPTVVGIPDGTAVVGRSFRVSIPTDLIASSGEIIKVSAAGKEALPSWLHWD
Bt              SALSDLHETVPTVVGIPDGTAVVGRSFRVTIPTDLIASNGEVIKVSAAGKEALPSWLHWD
Clf             SVLSDLHEAVPTVVGIPDGIAVVGRSFRVTIPMDLIASNGELVKVSAVGKEVLPSWLHWD
Ss              SVLSDLHEAVPTVVGIPDGTAVVGRSFRVTIPTDLIASGGEIIKVSAAGKEALPSWLHWD
Hs              SVLSDLHEAVPTVVGIPDGTAVVGRSFRVTIPTDLIASSGDIIKVSAAGKEALPSWLHWD
                *.***::*:********** *********:** *****.*:::****.***.********

Mm              PHSHILEGLPLDTDKGVHYISVSAARLGANGSHVPQTSSVFSIEVYPEDHNEPQSVRAAS
Rn              PHSHILEGLPLDTDKGVHYISVSAARLGANGSHVPQTASVFSIEVYPEDHSEPQSVRAAS
Bt              PQSHTLEGLPLDTDKGVHYISVSAARLGANGSHVPQTSSVFSIEVYPEDHSEPQSLRAAS
Clf             PQSHTLEGLPLDTDKGVHYISVSATRLGANGSHVPQTSSVFSIEVYPEDHSEPQSVRAAS
Ss              PQSHTLEGLPLDTDKGVHYISVSAARLGANGSHVPQTSSVFSIEVYPEDHSEPQSVRAAS
Hs              SQSHTLEGLPLDTDKGVHYISVSATRLGANGSHIPQTSSVFSIEVYPEDHSELQSVRTAS
                .:** *******************:********:***:************.* **:*:**

Mm              SDPGEVVPSACAADEPVTVLTVILDADLTKMTPKQRIDLLNRMQSFSEVELHNMKLVPVV
Rn              SDPGEVVSSACAADEPVTVLTVILDADLTKMTPKQRIDLLNRMQSFSEVELNNMKLVPVV
Bt              PDPGEVVSLVCAADEPVTVLTVILDADLTKMTPKQRIDLLRRMRGFSEVEPHNMKLVPVV
Clf             PDPAEVVSSACAADEPVTVLTVILDADLTKMTPKQRIDLLHRMRSFSEVELHNMKLVPVV
Ss              PDPGEVVSSVCAADEPVTVLTVILDADLTKMIPKQRLDLLQRMQSFSEVELHNMKLVPVV
Hs              PDPGEVVSSACAADEPVTVLTVILDADLTKMTPKQRIDLLHRMRSFSEVELHNMKLVPVV
                .**.***. .********************* ****:***.**..***** :********

Mm              NNRLFDMSAFMAGPGNAKKVVENGALLSWKLGCSLNQNSVPDIRGVETPAREGAMSAQLG
Rn              NNRLFDMSAFMAGPGNAKKVVENGALLSWKLGCSLNQNSVPDIRGVETPAREGTMSAHLG
Bt              NNRLFDMSAFMAGPGNAKKVVENGALLSWKLGCCLNQNSVPDIRGVEVPAREGAMSAQLG
Clf             NNRLFDMSAFMAGPGNAKKVVENGALLSWKLGCSLNQNNVPDIHGVEAPAREGAMSAQLG
Ss              NNRLFDMSAFMAGPGNAKKVIENGALLSWKLGCSLNQNSVPDIHGVEVPAREGAMSAQLG
Hs              NNRLFDMSAFMAGPGNAKKVVENGALLSWKLGCSLNQNSVPDIHGVEAPAREGAMSAQLG
                ********************:************.****.****.***.*****:***:**

Mm              YPVVGWHIANKKPTLPKRLRRQIH
Rn              YPVVGWHIANKKPTLPKRIRRQIH
Bt              YPVVGWHIANKKPSLPKRIRRQIH
Clf             YPVVGWHIANKKPPIPKRIRRQIH
Ss              YPVVGWHIANKKPPLPKRIRRQIH
Hs              YPVVGWHIANKKPPLPKRVRRQIH
                *************.:***:*****
```

Table ~~S2~~: Sequences alignment of hα-DG-Nt (residues 52-315) with selected sequences of mammalian α-DG-Nt region. Mm= Mus_musculus, Rn= Rattus_norvegicus, Bt= Bos_taurus, Clf= Canis_lupus_familiaris, Ss= Sus_scrofa, Hs= Homo_sapiens. Amino acid sequences were aligned in MUSCLE 3.8.

A B

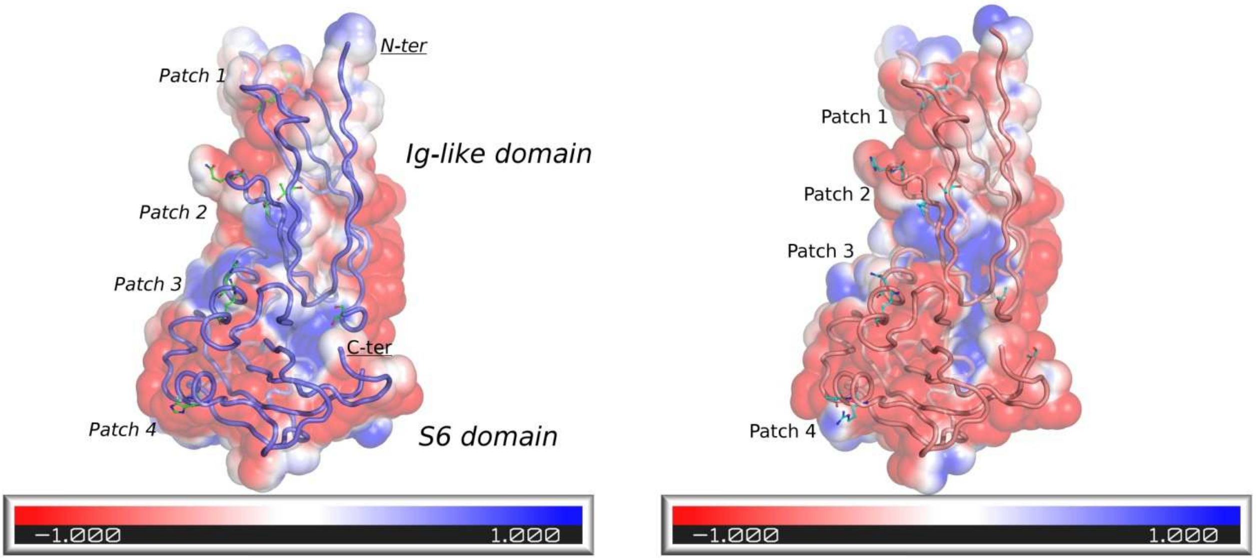

Figure S1. Electrostatic potential maps of mα-DG-Nt and hα-DG-Nt.
The electrostatic potential (k_bT/e_c) is mapped on the human and murine α-DG-Nt accessible surfaces. Molecular models are represented as ribbons. The human and the murine models are colored in blue and in pink-salmon respectively. A) hα-DG crystal structure. B) mα-DG-Nt crystal structure (PDB ID: 1U2C).

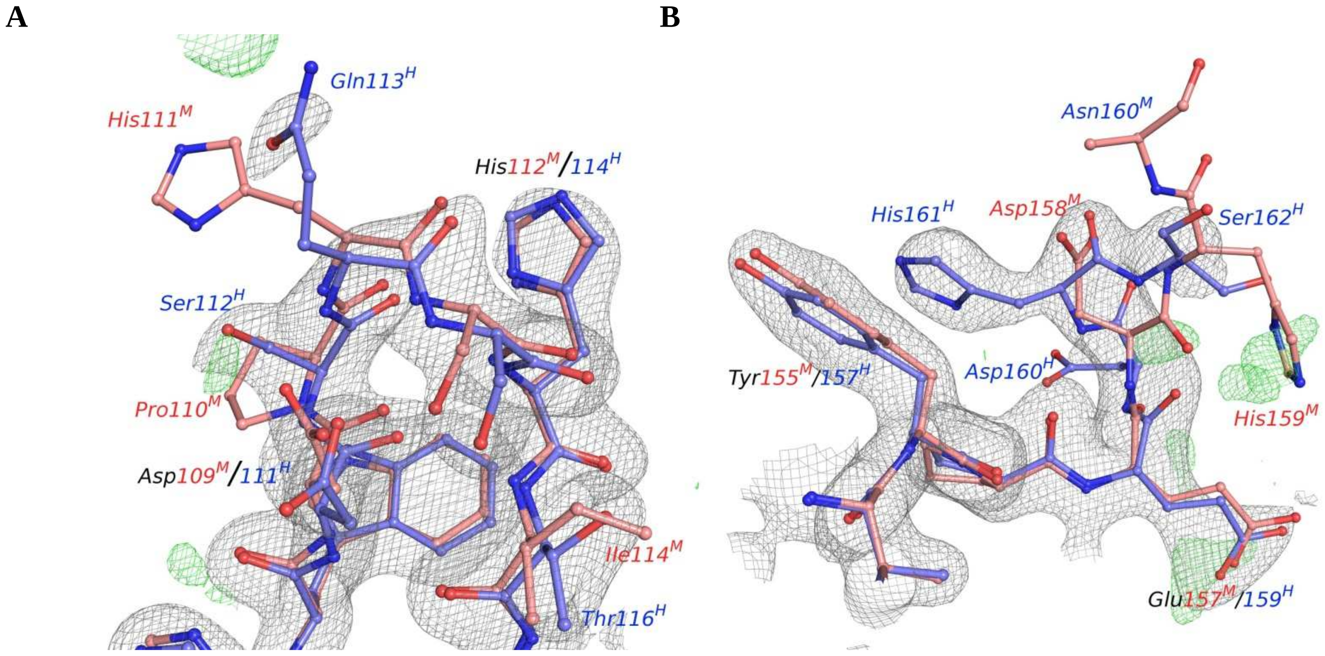

Figure S2. Superimposition of the hα-DG-Nt and mα-DG-Nt selected regions.
The models are represented as stick-and-ball, with mα-DG-Nt (PDB ID: 1U2C) colored in pink-salmon and hα-DG-Nt colored in blue. hα-DG-Nt residues are labelled with H superscript and mα-DG-Nt residues are labelled with M superscript. The models are overlaid by hα-DG-Nt σ_A-weighted $2F_o$-F_c map (contoured at 1.0 σ and colored in grey) and the F_o-F_c map (contoured at 3.0 σ and colored in green). A) 112-116 stretch (Patch P2). B) linker stretch (residues 157-162).

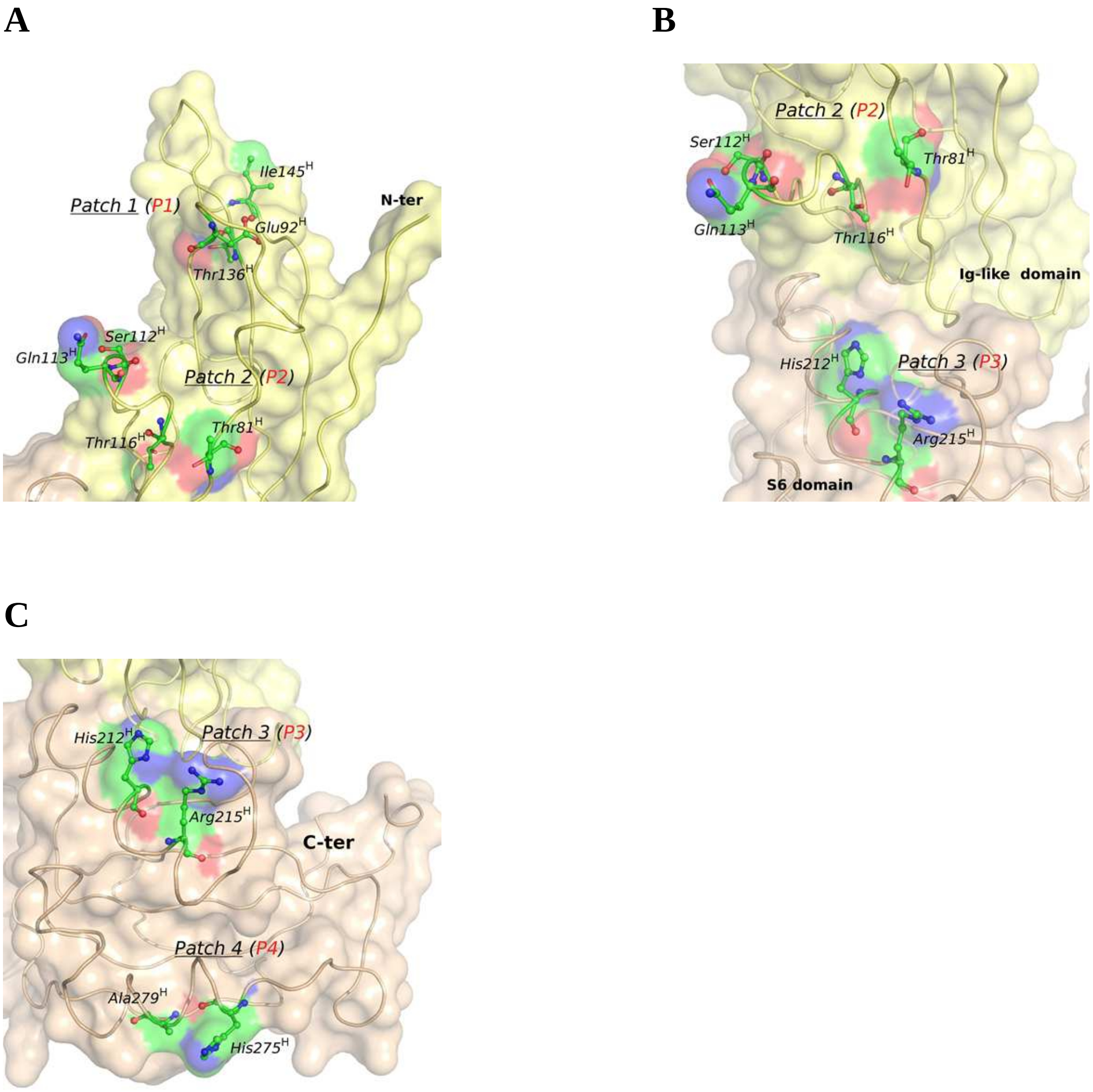

Figure S3. P1-P4 mapping on hα-DG-Nt accessible surface.

The hα-DG-Nt model is represented as ribbon, with the Ig-like domain colored in pale-yellow and the S6 domain colored in pale-pink. Residues belonging to the patches P1-P4 are depicted as a stick-and-ball model and their solvent accessible surfaces are mapped onto the hα-DG-Nt accessible surface. Residues and surfaces belonging to the patches are colored according to their atomic species. A) Patches P1 and P2. B) Patches P2 and P3. C) Patches P3 and P4.

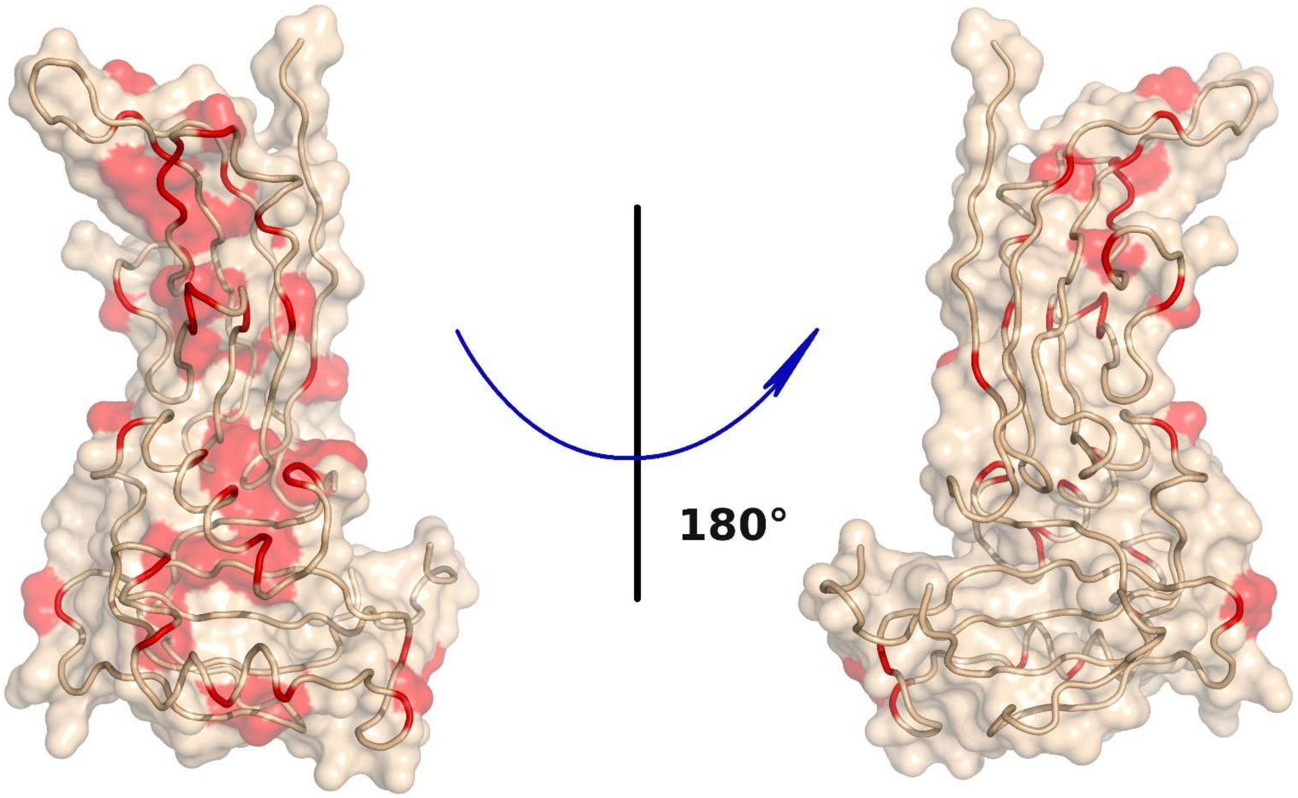

Figure S4. Amino acid differences mapped onto hα-DG-Nt accessible surface.

The hα-DG-Nt model is represented as ribbons. Amino acid differences based on the sequences alignment reported in Table ~~S1~~, are mapped onto the hα-DG-Nt accessible surface.
